# Cell mechanics and cell-cell recognition controls by Toll-like receptors in tissue morphogenesis and homeostasis

**DOI:** 10.1080/19336934.2022.2074783

**Published:** 2022-05-17

**Authors:** Daiki Umetsu

**Affiliations:** Graduate School of Life Sciences, Tohoku University, Sendai, Japan

**Keywords:** Toll-like receptors, planar polarity, myosin II, cell-cell adhesion, cell recognition, tissue morphogenesis, cell competition, cell mechanics

## Abstract

Signal transduction by the Toll-like receptors (TLRs) is conserved and essential for innate immunity in metazoans. The founding member of the TLR family, *Drosophila* Toll-1, was initially identified for its role in dorsoventral axis formation in early embryogenesis. The *Drosophila* genome encodes nine TLRs that display dynamic expression patterns during development, suggesting their involvement in tissue morphogenesis and homeostasis. Recent progress on the developmental functions of TLRs beyond dorsoventral patterning has revealed not only their diverse functions in various biological processes, but also unprecedented molecular mechanisms in directly regulating cell mechanics and cell-cell recognition independent of the canonical signal transduction pathway involving transcriptional regulation of target genes. In this review, I feature and discuss the non-immune functions of TLRs in the control of epithelial tissue homeostasis, tissue morphogenesis, and cell-cell recognition between cell populations with different cell identities.

## Introduction

**Figure 1.**
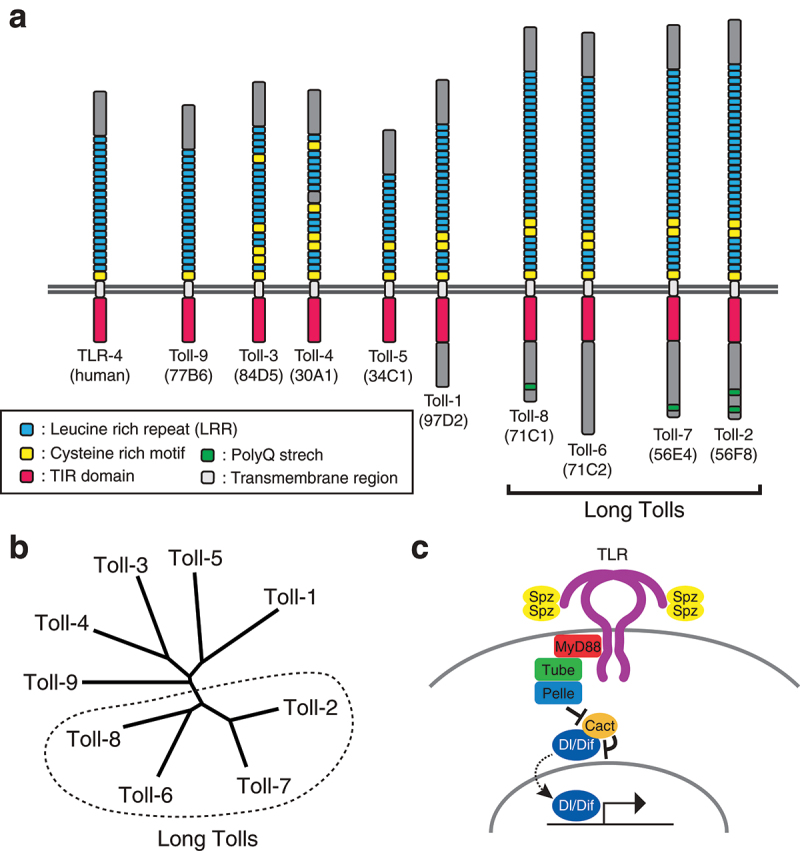
Toll-like receptors in *Drosophila* a) Domain structures of nine *Drosophila* TLRs. The clade of TLRs that have common features characteristic to insect TLRs, defined as long Tolls [36], is indicated with a bracket. b) Phylogenetic relationship of *Drosophila* TLRs. Toll-9 is the closest *Drosophila* TLR to vertebrate TLR. The long Toll clade is indicated with a dashed line. c) The canonical signal transduction pathway of Toll-1. Upon binding to the active ligand Spätzle (Spz), Toll-1 is activated and its conformational change leads to the recruitment of the adapter protein MyD88 through interaction with the TIR domain, which is present both in Toll-1 and MyD88. Once MyD88 binds to Toll-1, it forms a protein complex with Tube and Pelle, which then degrades the IκB protein Cactus (Cact), subsequently inhibiting the NFκB transcription factor Dorsal (Dl) and Dorsal related immunity factor (Dif) when Toll-1 is not activated. After the degradation of Cact, Dl/Dif is released to translocate into the nucleus where it promotes transcription of its target genes.

The *Drosophila* Toll-like receptor (TLR) protein family is a group of conserved proteins involved in numerous biological processes including pattern formation, innate immunity, cell competition, neuronal cell survival/death, wound healing, and tissue morphogenesis [1–13]. Toll (Toll-1) is the founding member of the genes encoding TLRs and was initially identified as a gene regulating dorsoventral patterning of the *Drosophila* embryo through the activation of the nuclear mediator Dorsal [14–17]. Toll-1 has leucine-rich repeat (LRR) domains flanked by characteristic cysteine-rich motifs in the extracellular domain and the Toll/Interleukin-1 receptor (TIR) domain in the intracellular domain, with a single transmembrane domain in between [4,18,19] (Figure 1(a)). 18-wheeler (Toll-2) was identified as a Toll-like receptor having a similar domain structure both in the extracellular and intracellular domains [20, 21]. Later, genome wide searches identified 7 more members named Toll-3 through Toll-9 in the fly genome [22,23] (Figure 1(a,b)). Toll-1 and Toll-7 are the only TLRs that have been shown to induce an immune reaction in flies, though the involvement of Toll-7 in the response to viral infection remains controversial [7,24–28]. The identification of Toll-1 as a receptor that stimulates innate immune responses in flies led to the cloning of a TLR in mammalian cells and the characterization of its role in the induction of the innate immune response [29]. Since then, numerous works have revealed the innate immunity functions of the TLRs, resulting in a thorough rendering of the overall picture of mammalian innate immune systems in response to diverse pathogen-associated molecular patterns (PAMPs) which TLRs directly recognize [30,31]. The canonical TLR signal transduction pathway is highly conserved between flies and vertebrates [1,8,32]. In *Drosophila* cells, the *Drosophila* NF-κB protein Dorsal (Dl) or Dorsal-related immunity factor (Dif), is bound to the IκB-like inhibitor Cactus (Cact) and sequestered in the cytoplasm in the absence of Toll-1 activation [1,16]. The activation of Toll-1 upon binding to the active Spätzle (Spz) ligand results in the recruitment of an adaptor protein complex consisting of MyD88, Tube, and Pelle [33]. The formation of the complex leads to the autophosphorylation of Pelle, which subsequently dissociates from the complex and degrades Cact, resulting in the release of Dl or Dif, which translocates into the nucleus and activates transcription of target genes [33](Figure 1(c)). In mammalian cells, counterparts to all these cytoplasmic components exist and transduce signal in a largely similar manner in their host defence system [1,8].

Phylogenetic analyses show that the *Drosophila* TLRs are more closely related to each other than they are to mammalian TLRs [34,35]. All insect species studied have largely the same set of directly corresponding TLRs, which suggests that the expansion of insect TLRs occurred in a common ancestor of the insect species [35]. It has been shown that *Drosophila* TLRs display a dynamic expression pattern during development [22] and indeed play important roles in various developmental processes beyond their well-known roles in dorsoventral axis determination through the conserved signal transduction pathway. A bioinformatic analysis of TLRs in *Tribolium* and other insect species showed that the members of the clade, in which Toll-2, 6, 7, and 8 are included, share a common feature: they are longer and encode more LRR repeats than those outside the clade (see Figure 1(a,b)). Accordingly, this clade has been termed the long Toll clade [36]. The *Drosophila* long-Tolls act together in embryonic axis elongation, and this morphogenetic function of TLRs is widely conserved among arthropods, highlighting the importance of TLRs in insect development [11,36]. In this review, I will provide an update on the diverse functions of *Drosophila* TLRs in development and non-immune functions, focusing primarily on recent progress in the areas of cell competition, neural network development, and epithelial morphogenesis, all of which depend on cell-cell recognition.

### Signalling through Toll-like receptors in cell competition

**Figure 2.**
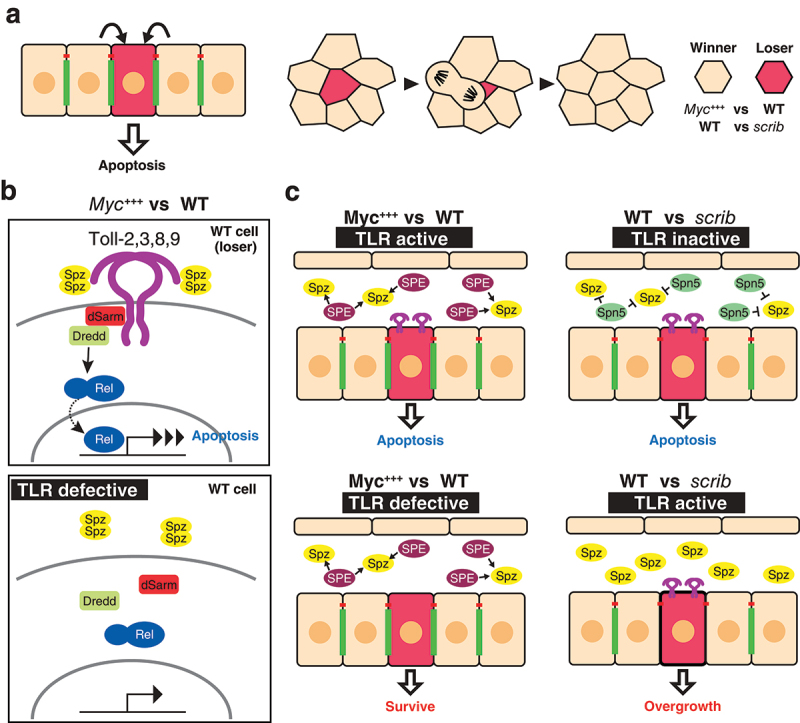
Toll-like receptors in cell competition a) The recognition of unfit cells (red cell) leads to the induction of apoptosis in epithelia (left). Once unfit cells are detected, they become loser cells and do not contribute to the tissue (right). In the case of Myc-induced cell competition, Myc-overexpressing cells become winner cells and wild type cells become loser cells. *scribble* (*scrib*) mutant clones become loser cells when surrounded by wild type cells. b) Signal transduction components of *myc*-induced cell competition. TLR signalling is upregulated in wild type loser cells, leading to the induction of apoptosis (top). When loser cells are defective for the transduction of TLR (e.g. loss of function of TLRs), wild type cells can survive (bottom). c) The competitive context induced by *myc*-induced cell competition (left). In *myc*-induced cell competition, the tissue is in the TLR signal activation-prone condition. Extracellular serine proteases such as Spz processing enzyme (SPE) are secreted into the lumen, and the TLR ligand Spz, which is present in the lumen, is activated, resulting in the activation of TLR signalling in wild type loser cells (top). When loser cells are defective for TLR signalling, wild type cells do not become loser cells and can survive (bottom). The competitive context induced by *scrib*-induced cell competition (right). The TLR ligand inhibitor Serpin 5 (Spn5) is secreted in this context. Due to the loss of TLR signalling, *scrib* mutant cells undergo apoptosis (top). When the cells are defective for *Spn5, scrib* mutants receive the active Spz and undergo the activation of TLR signalling, leading to the tumorigenic phenotype caused by over-proliferation (bottom).

Recent studies have identified TLRs as important regulators of the quality control mechanism cell competition, which maintains tissue integrity by inducing apoptosis in unfit cells and preventing them from contributing to the tissue [10]. It has been revealed in *Drosophila* epithelial tissues that cells intrinsically possess an ability to monitor the relative health states of their neighbours, and once suboptimal cells (those that are defective in protein synthesis, proliferation, or polarity) are detected, they become ‘loser cells’ that undergo apoptosis and do not contribute to the tissue [37,38](Figure 2(a)). *Minute* genes, which encode ribosomal proteins, and the oncogenic transcription factor Myc are the best-studied genes that induce competitive signalling between neighbours in developing epithelia. While *Minute* cells become loser cells and are removed from the tissue when surrounded by wild type cells, cells expressing more copies of *Myc* become super-competitors that induce cell death in neighbouring wild type cells [39–41]. Numerous genes that regulate this quality control system have been identified, yet the mechanism by which the cells recognize fit or unfit cells remains unclear [37,38]. TLR signalling is one of the signalling pathways that is activated under competitive conditions [10]. Loser cells upregulate TLR signalling when in contact with winner cells. Among nine *Drosophila* TLRs, Toll-1, 2, 3, 8, and 9 are crucial for the upregulation of the TLR signalling and the elimination of loser cells in the *Myc*-induced competition, while only Toll-3 and 9 are involved in the elimination of the *Minute* cells [10,42]. Interestingly, while the expression of Toll-8ΔLRR, a constitutively active form of Toll-8 [23], enhances the elimination of loser cells, it has no effect on control clones under non-competitive conditions, which suggests the requirement of additional components that are activated specifically in competitive contexts [10]. One possible model is that the heterotypic interactions of Toll-8 with Toll-1, 2, 3, or 9 are required for activating signal transduction. The activation of TLR signalling in cell competition requires both the ligand and downstream NF-κB nuclear factors. In the competitive context in the wing disc, the active form of the TLR ligand Spz becomes abundant due to the local production of the ligand by winner cells [42](Figure 2(c)). The involvement of TLRs in cell competition highlights an intriguing link between developmental control and innate immunity. The clonal growth disadvantage induced by TLR activation inversely correlates with the level of infection [43]. While the loser cell elimination is efficiently suppressed by reducing TLR signalling under normal conditions, the loser cell elimination becomes less reliant on TLR upregulation under axenic conditions. Therefore, a pro-apoptotic and anti-proliferative function of TLR signalling contributes to balancing the trade-off between clonal growth during development and innate immunity. The spatial regulation of the production of Spz may be a key to link these processes. While the lumen of the wing disc provides the TLR activation-prone environment in *Myc*-induced cell competition, that of the eye-antenna disc, where cell competition is *scrib*-induced, is a TLR suppressive environment [44](Figure 2(c)). *scrib* is a conserved apico-basal polarity gene whose loss results in tumourous overgrowth characteristic of neoplastic tumours [45]. Cells mutant for *scrib* and other neoplastic tumour-suppressor genes are eliminated by cell competition when surrounded by wild-type cells [46]. Both winner and loser cells secrete a negative Toll regulator Serpin, Spn5, to suppress aberrant TLR signalling activation in *scrib* cells in the competitive context [44]. The *scrib* cells are more sensitive to the loss of the TLR inhibitor and therefore undergo tumorigenic growth through the upregulation of the TLR signalling when Spn5 is reduced. In both the *Myc*- and the *scrib*-induced competition, misregulation of TLR signalling leads to the overgrowth of abnormal cells.

As mentioned above, the regulation of TLR is ligand dependent in the context of cell competition. The Spz ligand is structurally similar to mammalian neurotrophins (NTs), such as nerve growth factor (NGF), that promote neuronal cell survival, differentiation, axon targeting, connectivity, and programmed cell death [47,48]. Both Spz and mammalian NTs have a conserved cysteine-knot domain, form a disulphide-linked dimer, and undergo cleavage in the extracellular space to become their active forms [48,49]. Although mammalian NTs have a different set of receptors than TLRs, the signal transduction through the NT receptors also results in the activation of NF-κB [50,51]. Based on these notions, the *Drosophila* TLR was expected to transduce signal in the regulation of the neuronal survival and death in a ligand dependent manner. In fact, in the *Drosophila* nervous system, Toll-6 and 7 have been shown to regulate cell survival using DNT1 and DNT2, respectively [9]. It has been also shown that distinct adaptors downstream of Tolls can drive either apoptosis or cell survival [3]. Changes in spatial and temporal patterns of adapter distribution segregate the distinct neural circuits. A more recent report suggests that Toll-6 acts in glial cells but not in neuronal cells to remove apoptotic debris of neurons in the central nervous system [52]. Toll-6 is activated by DNT2 produced and processed by apoptotic neurons and the activated Toll-6 upregulates phagocytotic genes. As a consequence, impaired glial Toll-6 signalling causes early onset of neurodegeneration. Together with the context dependent dual role of TLRs in regulating both the apoptotic cell death and the overgrowth of aberrant cells in the cell competition, balancing cell survival and death in the central nervous system highlights an interesting common function of the TLR signalling in the homeostatic regulation of cell number in distinct tissues and in both post-mitotic and proliferating cells.

### Toll-like receptors in germband elongation

**Figure 3.**
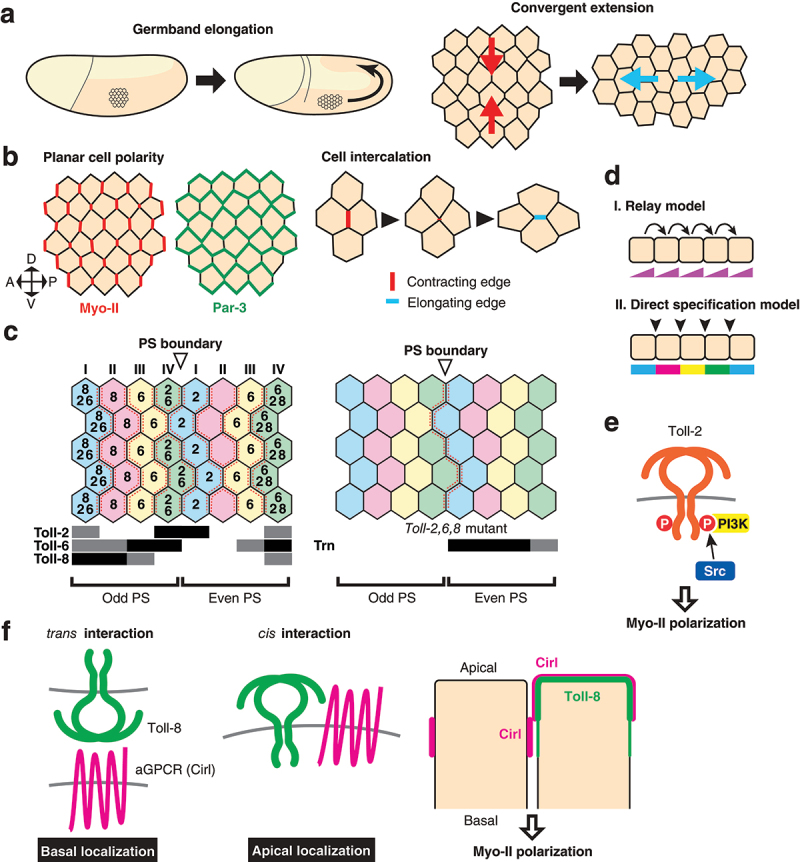
Toll-like receptors in the control of germband elongation a) Germband elongation of the Drosophila embryo. Embryos at stage 6 to 9 are illustrated (left). Cells that undergo convergent extension are depicted (right). Arrows indicate the direction of tissue deformation. b) Molecules and cellular mechanism of cell intercalations. Myosin II (Myo-II) is enriched on the anterior and posterior cell edges throughout the tissue, while Par-3 is enriched on the dorsal and ventral edges for each cell (left). Contraction of a vertical edge followed by the formation of the 4-way vertex and elongation of a horizontal edge after the junctional exchange results in the cell intercalation (right). c) Spatial expression patterns of TLRs. Numbers within the cells indicate TLRs expressed. Horizontal bars below the schematic indicate expression domains for referenced TLRs (left). Odd and even parasegments (PS) are indicated. Myosin-II accumulation is indicated by dashed red lines. Myo-II is still present in Toll-2,6,8 triple mutants (right). The Leucine rich repeat protein Tartan (Trn) is expressed in even parasegments. d) Two possible models of planar cell polarity. In the relay model (i), arrows depict the signal from a neighbour on one side is relayed to the neighbour on the other side. Protein activity gradients are established in each cell, resulting in the robust transmission of polarity information from one to another. The direct specification model (II) does not transmit signal from one cell row to next. The differential cell identity between neighbours is sensed directly and polarizes the cell. e) Signal transduction by Toll-2 in the regulation of planar polarity at the edge of the Toll-2 expression domain. f) Interaction of Toll-8 with the adhesion GPCR Cirl in *trans* and *cis. trans* interaction leads to a basal shift of Cirl localization, while *cis* interaction leads to an apical shift. The resulting asymmetry of Cirl localization leads to the recruitment of Myo-II at that cell junction.

The TLR signalling pathway was initially identified in embryonic dorsoventral axis formation [8]. However, TLRs play developmental roles in tissue patterning, not only through transcriptional regulation but also in the mechanical regulation of cell-cell contacts independent of transcriptional regulation. Germ-band elongation is one of the main morphogenetic movement during *Drosophila* gastrulation and constitutes a powerful model to understand the mechanism by which local cell behaviour drives tissue scale morphogenesis. The embryonic epidermis, ‘the germband’, contracts along the dorsoventral axis and doubles in length along the anteroposterior body axis through a process called convergent extension, a conserved tissue-scale morphogenesis in animals [53–56](Figure 3(a)). Cells undergo oriented intercalations as a consequence of junctional remodelling that exchanges neighbours [57,58](Figure 3(a,b)). The actin binding motor protein non-muscle Myosin II (Myo-II) is localized at cell edges on the anterior and posterior side of the individual cells (Figure 3(b)). The planar polarized Myo-II localization on cell edges is critical for the directed cell intercalation during convergent extension because it drives the contraction of the vertical (AP) edges, which are followed by neighbour exchanges, and thereby directs cell intercalations throughout the epidermis [57,59–61](Figure 3(b)). The multi-PDZ domain protein Par-3 localizes at the cell cortex of the horizontal (DV) edges, excludes Myo-II, and stabilizes cell-cell adhesion at these edges [61,62](Figure 3(b)). Therefore, the planar polarity of the epidermal cells plays a crucial role in the oriented cell intercalations during germband elongation. How do these cells acquire planar polarity in this tissue? Paré *et al*. found that TLRs act as key regulators for planar polarity establishment, cell intercalations, and germband elongation [11]. Toll-2, 6, 7, 8 are expressed in overlapping stripes perpendicular to the body axis under the control of the pair-rule genes *eve* and *runt* [11,22](Figure 3(c)). Embryos lacking Toll-2, 6, 8 display reduced axis elongation, similar to *eve* and *runt* mutants. Cell intercalation is also defective in *Toll-2, 6, 8* triple mutants, and more strikingly both Myo-II and Baz planar polarity are greatly reduced. There is currently no clear answer for how the multiple TLRs establish planar cell polarity at each cell row. One model proposes that the multiple TLRs cooperatively act to establish planar polarity by forming a Toll code with their partially overlapping expression patterns [11]. This view is supported by the experimental evidence that the TLRs bind to each other in a heterophilic manner. Toll-2 expressed in cultured S2 cells preferentially binds to the extracellular domain of Toll-8 rather than that of Toll-2, suggesting heterophilic binding between Toll-2 and Toll-8. Moreover, an array of co-culture aggregation assays using cells expressing TLRs have shown that cells expressing Toll-2 form extensive heterophilic contacts with cells expressing Toll-6 and/or Toll-8. These results support a model in which each cell row that expresses the same set of TLRs in the embryonic epidermis can physically interact with their anterior- and posterior- adjacent neighbours expressing a different set of TLRs. These heterotypic junctions formed by neighbouring cells with different sets of TLRs create a unique platform that allows for the accumulation of more actomyosin than at the DV edges. However, how the heterotypic interactions signal to initiate actomyosin reorganization remains unknown. In an alternative model, each boundary of the TLR expression domain may planar polarize cells independent of interaction with other TLRs. This model is supported by detailed analyses of Toll-8 [6] that demonstrate, among other findings, that ectopic Toll-8 expression in the absence of endogenous Toll-2, 6, 8 is sufficient to induce recruitment of Myo-II at the border of the ectopic expression domain, which suggests that the Myo-II enrichment on cell junctions is established solely by the differential expression level of Toll-8 and does not require interaction with other TLRs.

Another interesting consideration is how planar polarity is established at single cell resolution. There are two possible mechanisms through which planar polarity can be achieved in each cell (Relay model and Direct specification model, Figure 3(d)). The first, the relay model, is adopted by the planar cell polarity (PCP) system mediated by Frizzled (Fz) and its interacting proteins. Planar polarity is typically observed in wing hairs, bristles, and ommatidial alignment in *Drosophila* epithelia. These structures are oriented by the Fz-dependent core PCP signalling system in which the signals are relayed from one cell to another through direct cell-cell contacts (Relay model, Figure 3(d))[63–66]. Interestingly, the mechanism that controls planar polarity during *Drosophila* germband elongation is not dependent on the Fz core PCP system [61], while the vertebrate counterparts are required for convergent extension movements during axis elongation [53,55,67,68]. For example, the depletion of the PCP component, Clsr1, by siRNA in the chick neural plate results in defects of convergent elongation accompanied with the loss of polarized localization of active Myosin II [69].

The second mechanism by which planar polarity is achieved is the direct specification model (Figure 3(d)). In *Toll-2* null mutants, planar polarity specifically at the border of the *Toll-2* expression domain is lost, but that of the AP edges inside the domain is unaffected [12]. Similarly, in embryos with depleted *Toll-2, 6*, and *7*, where endogenous *Toll-8* alone is expressed, Myo-II remains accumulated along the stripes at the border of *Toll-8* expression domains [6]. Thirdly, after the completion of a series of oriented cell intercalations, some stripes of Myo-II enrichment disappear [70]. Each cell row that previously made contacts with another cell row expressing a different set of TLRs begins to make contacts with another row of cells that express the same set of TLRs [70]. At these homotypic contacts Myo-II is no longer enriched, which suggests that the differential expression boundary plays a key role in accumulating Myo-II. These results indicate that the polarity cue does not have to be relayed globally throughout the entire length of the epidermis to establish planar polarity, but rather it can be established locally, independent of the transmission of signal cues from one cell row to another (see Direct specification model, Figure 3(d))[70]. The overlapping TLR expressions individually specify the identity of stripes of junctions at a one cell-wide resolution. This mechanism assumes that there are a series of cell surface molecules that specify individual stripes of junctions with a unique identity encoded by cell surface molecules that are expressed differentially at the single cell row resolution. However, the Toll code is incomplete and, for example, cannot explain Myo-II enrichment at parasegmental boundaries to support the direct specification model [11,70]. By considering the expression pattern of TLRs in the embryonic epidermis, numerical analysis predicts that in addition to the pair-rule patterns of Toll-2, 6/8 (6 and 8 are considered to be the same pattern), a third receptor should be expressed in every other parasegment [70]. Consistent with this observation, the cells that are in contact with parasegmental boundaries still display strong planar polarity in *Toll-2, 6, 8* mutant embryos (Figure 3(c)). It was subsequently shown that the expression of another LRR protein, Tartan (Trn), provides a regional cue to polarize Myo-II at parasegmental boundaries. Trn is expressed in even number parasegments and is crucial for the planar polarity of Myo-II and Par-3 at parasegmental boundaries due to its recruitment of the ubiquitously expressed transmembrane protein Ten-m [71]. Altogether, work in this area has shown that the mechanism for Myo-II and Par-3 planar polarity in *Drosophila* germband elongation is distinct from that of the Fz core PCP pathway that is used for the Myo-II planar polarity during convergent extension in vertebrate neural tube formation [69].

Given the localized activity of TLRs within cells and the rapid timescale of cell intercalations, it is likely that TLRs are engaged in a more direct action on cytoskeletal machineries than the transcriptional regulations in the germband elongation. The non-receptor tyrosine kinase Src42 localizes at cell junctions and is preferentially activated at the AP edges [12]. This planar polarized Src42 activation is dependent on *Toll-2, 6, 8*. Defects in active Src42 localization are evident even in *Toll-2* single mutants. Src42 promotes the phosphorylation of Toll-2 and 6 but not Toll-8. Phosphorylated Toll-2 recruits and activates the PI3K, which catalyzes the conversion of phosphatidylinositol (4,5)-bisphosphate into phosphatidylinositol (3,4,5)-trisphosphate, at the AP edges across which Toll-2 is differentially expressed [12](Figure 3(e)). Planar polarized PI3K is necessary for Myo-II and Par-3 planar polarity, oriented cell intercalations, and convergent extension. Despite uniform Toll-2 protein localization throughout the entire cell, active Src42 and PI3K are enriched specifically on the AP edges. The mechanism via which Src activation is confined to the AP edges remains unknown. Identifying the factor that triggers the activation of Toll-2 intracellular domain locally within cells will provide a missing link to bridge the gap between regional cell type specification by pair-rule genes and tissue morphogenesis.

Ectopic Toll-8 expression in mosaic clones in wing discs sorts out cells from surrounding wild type cells, and cell sorting is accompanied by Myo-II accumulation at clone borders [6,72]. Unlike Toll-2, which requires its intracellular domain to planar polarize Myo-II and Par-3, Toll-8 does not require its intracellular domain for Myo-II recruitment at the differential expression border. Somatic clones of cells overexpressing intracellular domain-lacking Toll-8 sufficiently enrich Myo-II at clone borders, suggesting the existence of another mechanism whereby Myo-II is recruited independently of the recruitment of PI3K. The extracellular domain of Toll-8 interacts with the adhesion G-protein coupled receptor Cirl [6]. Mosaic clone analyses show that Myo-II accumulates at the interface of the asymmetric expression of Cirl. Toll-8 regulates the localization of Cirl both in cell autonomous and non-cell autonomous manners (Figure 3(f)). Toll-8 interacts with Cirl in *cis* and redistributes Cirl more apically than its normal localization site. In addition, Toll-8 can interact with Cirl on neighbouring cells in *trans* and recruits Cirl at cell contacts more basally (Figure 3(f)). Toll-6, but not Toll-2, interacts with Cirl similarly to Toll-8. Therefore, differential expression of Toll-8 breaks the symmetry of the apicobasal localization of Cirl, which then leads to local accumulation of Myo-II at the boundary (Figure 3(f)). This work reveals an interesting feature of TLRs: despite their structural similarity, TLRs in the long Toll clade polarize Myo-II in a totally different manner from one another (Figure 1(a,b)).

### Role of Toll-like receptors in compartment boundary maintenance

**Figure 4.**
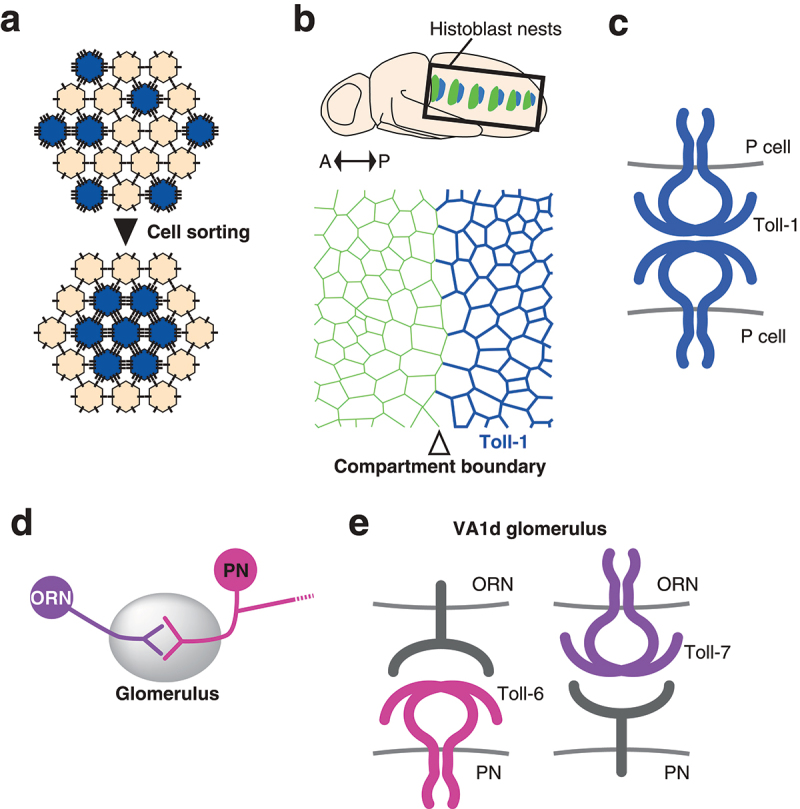
Cell-cell adhesion by Toll-like receptors in tissue morphogenesis and the wiring of the olfactory nervous system a) Differential adhesion model. Cells expressing more adhesion molecules (blue) replace the weaker adhesions with the stronger ones, resulting in the relocation of these cells inside the cell aggregate. b) Differential expression of Toll-1 in the histoblasts of the pupal abdomen. Locations of histoblast nests are indicated (top). Anterior to the left and posterior to the right. Toll-1 is strongly expressed in the posterior compartment (blue). Compartment boundary is indicated by the open arrowhead (bottom). c) Homophilic adhesion of Toll-1 between posterior compartment cells (P cells). d) Wiring between olfactory receptor neurons and projection neurons in the glomerulus. e) TLRs in the ORN-PN matching. In the VA1d glomerulus the pre-synaptic VA1d ORN and the post synaptic PN form a connection. Toll-6 functions in the PN and Toll-7 in the ORN. Toll-6 does not require the expression of Toll-7 in the cognate ORN and *vice versa*. Neither protein requires the intracellular domain, suggesting the presence of heterophilic binding partners.

Cells in vertebrate and invertebrate tissues are often subdivided into non-mixing territories called compartments that define lineage restriction boundaries [73]. The compartment boundary restricts the mixing of cell lineages and displays remarkably straight morphology [74–77]. One of the theories of cell sorting, the differential interfacial tension hypothesis, attributes cortical tension to a direct driving force for cell sorting [78,79]. Therefore, it is expected that cell sorting caused by mosaic Toll-8 misexpression is the consequence of increased mechanical tension due to Myo-II accumulation at the clone border [6,80–84]. On the other hand, the differential adhesion hypothesis, the longer-standing model, considers the difference in adhesiveness between two cell populations as the primary driver of cell sorting [85](Figure 4(a)). The structure of the Toll-1 extracellular domain was originally found to be similar to that of the human membrane receptor and adhesion factor platelet glycoprotein 1b (Gp1b) [4,18]. In fact, cell aggregation assays characterize Toll-1 as well as Toll-2 as a heterophilic adhesion molecules [18,21]. No binding partners have been identified for either protein so far. Although the distribution of maternal *Toll-1* RNA is uniform, zygotic *Toll-1* RNA displays dynamic spatial and temporal patterns in the embryo [86]. *Toll-1* is also expressed in the developing epidermal epithelium of the pupal abdomen, called histoblast nests [5](Figure 4(b)). In this tissue, cells are segregated into neighbouring anterior and posterior compartments [87–89]. Interestingly, Toll-1 is highly expressed in the posterior compartment, having a sharp expression boundary that coincides with the compartment boundary [5](Figure 4(b)). Tissues defective for *Toll-1* fail to maintain the sharp and straight compartment boundary, suggesting that *Toll-1* is responsible for maintaining the lineage restriction boundary [5]. Moreover, clones overexpressing Toll-1 display smooth borders like those found in Toll-8 overexpressing clones. While Toll-1 does not require its intracellular domain to induce cell sorting, Toll-1 clones do not accumulate Myo-II at clone borders, which implies the existence of a different mechanism from that of Toll-8. This finding also contrasts with the role of *Toll-1* in wound repair in embryos [2]: upon wounding the embryonic epidermis, cells at the wound edge assemble supracellular actin cables to close the gap efficiently. *Toll-1* mutants fail to form actin cables and close the wound. However, this process requires NF-κB genes, the effectors that regulate target gene transcription in the canonical Toll signal transduction pathway [2]. Detailed analysis of Toll-1 localization using mosaic clones in histoblasts reveals that Toll-1 acts as a homophilic adhesion molecule, showing yet another mode of TLR function involved in the regulation of mechanical interaction between cells [5]. The function of Toll-1 as a homophilic adhesion molecule has been shown by mosaic clone analyses in tissue. For example, in clones expressing a full-length *Toll-1* tagged with the Venus fluorescent protein (Toll-1-Venus), Toll-1 localizes at cell-cell contacts between cells expressing the Toll-1-Venus (cell-cell contact within clones) but not at the heterotypic cell-cell contacts between cells with and without Toll-1-Venus expression (cell-cell contacts at clone edges). Theoretically, increasing adhesion results in lowering tension at the corresponding cell-cell contacts [90]. Quantitative analysis of cell dynamics reveals the cellular level mechanism: the homophilic binding of Toll-1 *in trans* stabilizes the cell-cell contacts enabling them to become more resistant to separation caused by the fluctuation of cell area resulting from pulsatile actomyosin coalescence [5]. Toll-1 is highly expressed in the dorsal vessel and is thought to mediate physical interactions between cells during the closure of the dorsal vessel [91], which can be explained by homophilic binding activity. Alternatively, it is possible that Toll-1 acts as a heterophilic adhesion molecule that requires a yet-to-be-identified binding partner [18]. Of the other TLRs, Toll-5 is most closely related to Toll-1 and also has a similar expression pattern to Toll-1, including in the dorsal vessel [22], which suggests functional similarity, cooperation, or redundancy. It is, however, noteworthy to mention that although Toll-5 displays a largely overlapping expression pattern with Toll-1, especially in mesodermal tissues such as ventral muscles and the dorsal vessel, it is not expressed in epidermal tissues. This notion further supports the idea that Toll-1 does not require any binding partners in the histoblast nest. The function of TLRs as adhesion molecules may play more important roles in tissue organization that has previously been recognized.

### Role of Toll-like receptors in neuronal connectivity

Toll-1 is also expressed in muscles during embryogenesis and has a function in the precise connection and synapse formation of motoneurons with muscles [92]. Toll-1 prevents the ectopic connection between motoneurons and muscles, and this function may be achieved again by the homophilic or heterophilic binding activity that adheres juxtaposed muscles, which then blocks the ectopic innervation in between these muscle fibres. On the other hand, it is also possible that Toll-1 present on muscle fibres prevents ectopic innervation by interfering with synapse formation through the interaction with an unidentified binding partner that is present on the innervating neuron and transduces a signal to initiate the synapse formation [92]. Toll-6 and Toll-7 have also been found to act in neural connection; Toll-6 and 7 are expressed in the neurons of the olfactory system and play an essential role in wiring neural circuitry during brain development [13]. Olfactory circuit formation occurs in a structure called the glomerulus in the antennal lobe. The pre-synaptic neurons, olfactory receptor neurons (ORNs), extend their axons to the antennal lobe and target a single invariant glomerulus depending on their identity, which is specified by the expression of a particular olfactory receptor gene [93–95]. The post-synaptic neurons, projection neurons (PNs), arborize dendrites within a single glomerulus and connect with the innervating axons of the corresponding ORNs [96,97] (Figure 4(d)). Toll-6 is expressed in subsets of both ORNs and PNs, acting mainly in PN dendrite targeting [13]. Toll-7 is expressed in a subset of ORNs and functions in the proper targeting of their axons and matching with corresponding PNs. DNT1 and DNT2, which are known ligands for Toll-6 and 7 that play roles in neuronal survival and death (see above) [3,9], are dispensable for the matching of ORN axons and PN dendrites. Toll-6 and Toll-7 are not ligands of each other, and their activity in the specific ORN-PN partner matching is proposed to be dependent on interaction with an unidentified heterophilic ligand [13](Figure 4(e)). The extracellular but not the intracellular domain is utilized for wiring specificity for both Toll-6 and Toll-7, suggesting that they serve as a cell-surface tag protein to ensure proper cell-cell recognition [13]. Toll proteins have putative glycosylation sites and have been experimentally shown to be N-glycosylated [98,99]. Therefore, it is possible that the modification of the extracellular domain of Toll-6 and Toll-7 is also involved in altering the selectivity of ORN-PN matching. Interestingly, an endoplasmic reticulum (ER) resident protein, Meigo which is a homologue of mammalian UGTrel1 that transports nucleotide sugars required for glycosylation reactions in the ER, was identified as a molecule required for proper PN dendrite targeting like Toll-6 [100]. Meigo-mediated N-glycosylation of the transmembrane protein Ephrin is essential for the dendrite refinement of PNs. However, *ephrin* mutants do not recapitulate another severe mistargeting phenotype of *meigo* mutant PN dendrites, which suggests the presence of another Meigo-modified cell surface protein. Given that TLRs are N-glycosylated, it is expected that Toll-6, whose mutation shows a similar phenotype to that of *meigo* mutants, may require Meigo-mediated N-glycosylation for optimizing its ability to target the PN dendrite properly. In fact, a recent report has shown that Toll-6 is N-glycosylated and that the expression level of Toll-6 is regulated by *meigo* in cultured cells [101]. Furthermore, dendritic Toll-6 localization is altered in *meigo* mutant PNs [101]. These results highlight the importance of the biochemical modification of TLRs in their roles in neuronal connection and potentially other systems.

## Conclusion

Recently, novel functions of TLRs in the direct control of mechanical properties such as cell contractility or cell-cell adhesion during tissue morphogenesis have been revealed in *Drosophila* tissues. These mechanical regulations are implemented by interaction with other molecular machineries that cooperate to recruit contractile Myo-II independent of the TIR domain, which has an essential role in activating the NF-κB transcription factors [6,12]. However, the mechanisms that link the TLR interacting proteins with Myo-II have yet to be identified. Symmetry breaking within the cell is a fundamental process that is crucial for driving tissue morphogenesis [102]. The study of Toll-8, which induces the asymmetry of the aGPCR Cirl along the apicobasal axis of cells, reveals a novel symmetry breaking system for the planar polarity of epithelial cells. It will be interesting to see in the future how asymmetrically localized Cirl is activated and how it signals intracellularly to recruit Myo-II. Toll-2 interacts with PI3K whose effectors are known to regulate the actomyosin cytoskeleton [103,104]. However, how Toll-2 is activated only at the interface between cells expressing and non-expressing cells remains unknown. Whether this activity requires ligand binding or heterotypic interaction with other TLRs is an intriguing question that requires further investigation. Furthermore, there may be co-receptors for Tolls whose extracellular domain is sufficient to elicit their full functions. Again, it will be interesting to see in the future if these TLRs function through interactions with other TLRs or novel cell surface molecules. Modification of the extracellular portion may also play a role in modifying the binding specificity of TLRs with their partners. Identification of these molecular intricacies will provide new insight into the understanding of the molecular mechanisms that underlie tissue morphogenesis, including how different outputs utilize similar sets of molecular machineries.

NF-kB activation is required for the induction of cell competition downstream of an increase in autophagic activity, which is common to both the *Minute*-induced and *myc*-induced competition [105]. Autophagic puncta are preferentially observed at clone borders, which is difficult to explain solely by the action of the diffusive Spz ligand present in the extracellular lumen. Therefore, it is possible that the cell competition system requires local cell-cell interactions such as physical contact between winner cells and loser cells in addition to the ligand-dependent NF-kB activation system that induces apoptosis. Since TLRs have been shown to act as adhesion molecules both *in vitro* and *in vivo*, heterotypic or homotypic adhesion of TLR in *trans* may help promote the interaction between winner cells and loser cells in the context of cell competition.

Future work will elucidate the important unestablished roles of TLRs in tissue-scale phenomena and identify potentially common principles of the TLR functions in their regulation of non-immune functions both in *Drosophila* and vertebrates. By integrating accumulating evidence for the physiological roles of TLRs obtained in various *Drosophila* systems, we also expect to discover novel principles that govern well-designed cell-cell interaction systems such as tissue morphogenesis and cell competition. The studies of TLR biology in *Drosophila* systems will continue to provide conceptual advances in understanding how cells interact and utilize molecular machineries in the context of development and tissue homeostasis under physiological as well as pathological conditions.

## Data Availability

No datasets were generated or analyzed as a part of this work.
